# Widespread retina and optic nerve neuroinflammation in enucleated eyes from glaucoma patients

**DOI:** 10.1186/s40478-022-01427-3

**Published:** 2022-08-19

**Authors:** Carola Rutigliani, James R. Tribble, Anna Hagström, Emma Lardner, Gauti Jóhannesson, Gustav Stålhammar, Pete A. Williams

**Affiliations:** 1 Department of Clinical Neuroscience, Division of Eye and Vision, St. Erik Eye Hospital, Karolinska Institutet, 171 64 Stockholm, Sweden; 2 St. Erik Eye Hospital, 171 64 Stockholm, Sweden; 3 Department of Clinical Sciences, Ophthalmology, Umeå University, 901 85 Umeå, Sweden; 4 Wallenberg Centre of Molecular Medicine, Umeå University, 901 85 Umeå, Sweden

**Keywords:** Neuroinflammation, Retina, Glaucoma, Histopathology, Optic nerve, Microglia, Astrocytes

## Abstract

Neuroinflammation is recognized as a key component of neurodegenerative disease. In glaucoma, a common neurodegenerative disease and the leading cause of irreversible blindness, the evidence for neuroinflammation in patients is lacking. Animal models have demonstrated significant pro-inflammatory activation of resident glia in the retina, as well as influx of blood-derived monocytes and pro-inflammatory factors. Confirmation of this in human donor tissue has been challenging due to a lack of well-preserved and well-characterized post-mortem tissue. To address this we utilize archived, wax embedded eyes fixed immediately following enucleation from living glaucoma patients. We compared glaucoma to control eyes (enucleated for uveal melanoma where the tumor did not impact the central retina or optic nerve). We performed immunolabelling for neurodegenerative and glial markers (CD45, CD163, IBA1, GFAP, Vimentin) which were quantified by high-resolution light microscopy and image analysis in FIJI. Glaucoma eyes demonstrated significant neural loss consistent with advanced neurodegeneration. IBA1 and GFAP were significantly increased in the retina and optic nerve head of the glaucomatous eyes indicating that significant neuroinflammation had occurred which support findings in animal models. Inflammation is a treatable symptom of many diseases and as such, identification of earlier inflammatory processes in glaucoma could be important for potential future treatment options.

**Supplementary Information:**

The online version contains supplementary material available at 10.1186/s40478-022-01427-3.

## Introduction

Glaucoma is the leading cause of irreversible blindness affecting an estimated ~ 80 million people worldwide. With our increasingly aged population this burden will increase and is predicted to affect ~ 112 million people by 2040 [1]. In glaucoma, loss of vision occurs through the progressive death of retinal ganglion cells (RGCs), the output neurons of the retina. This manifests clinically as a thinning of the retinal nerve fiber layer (RNFL; RGC axons), the ganglion cell layer (GCL; RGC somas), and inner plexiform layer (IPL; RGC dendrites), and cupping/excavation of the optic nerve head (ONH; where RGC axons bundle and exit the eye as the optic nerve). Whilst the underlying mechanisms of glaucoma are still not fully understood, there is increasing evidence that the immune system may play a significant role in the disease [2]. Importantly, inflammation is a treatable symptom of many diseases and as such, identification of potential inflammatory processes that may occur in glaucoma could lead to viable, additive treatment opportunities for patients in the future.

The eye is often considered an immune privileged site [3]. However, an increasing number of studies have shown the importance of both resident and infiltrating immune cells in the initiation and progression of glaucoma. Animal models have demonstrated early and progressive pro-inflammatory activation of resident glia (microglia, astrocytes, and Müller glia), infiltration of monocytes and other blood leukocytes, up-regulation and release of cytokines/chemokines, especially the complement system, and activation of neurotoxic responses [4–10]. Targeting these processes directly has been demonstrated to delay or halt the neurodegenerative processes in glaucoma animal models. For example, the removal of systemic immune cells via gamma radiation, inhibition of microglia proliferation and activity (genetically or pharmacologically), and inhibition of the complement cascade [11–13].

A number of the neuroinflammatory features identified in animals have been demonstrated in human glaucoma patients. Histological studies of human retina have shown intense inflammatory staining in the ONH of glaucoma eyes [14], increased protein levels of pro-inflammatory cytokines (TNF-α, IL-1β, IL-6, IL-8, and IFN-γ [15]) and infiltration of CD163+ macrophages to the optic nerve [16]. In human glaucomatous ONHs, astrocytes also expressed high levels of TNF-α and TNFR [17] and there is increased expression of toll-like receptors on retinal microglia and astrocytes [18]. The presence of a *TNF*-308 G/A polymorphism which leads to greater transcriptional activation has been demonstrated to be significantly higher in primary open-angle glaucoma and pseudoexfoliative glaucoma in specific populations [19, 20]. While a number of SNPs with the potential to affect inflammation have been identified to increase risk of glaucoma, these have been experimentally investigated in the context of intraocular pressure (IOP) and anterior chamber pathology [21]. Analysis of blood, aqueous humor, and vitreous has demonstrated the increased presence of TNF-α, complement components, autoantibodies, and other signs of inflammation [22–28]. Increased deposition of complement component 1Q (C1q) and 3 (C3) in particular have demonstrated to be well conserved inflammatory signals across animal models and human glaucoma samples [10]. Genetic deletion or pharmacological inhibition of C1q in animal models protects retinal ganglion cells, whereas C3 deletion worsens retinal ganglion cell survival [13, 29] reflecting the complex and time specific role of inflammation in glaucoma and the need for targeted therapies over broad anti-inflammatory strategies.

To date, there have been no successful clinical trials that assess an anti-inflammatory strategy in glaucoma. A major hurdle is the paucity of high quality human data with which to corroborate findings in animal models. The neural retina degrades rapidly following death [30], and this will inevitably lead to neurodegeneration and neuroinflammation. Human retina at early disease time points with good tissue preservation are exceptionally rare. Linking donor-tissue to well established medical records for disease staging is also difficult (especially as follow-up intervals for established glaucoma patients may vary considerably). As such, whether blood derived immune cells infiltrate the retina in human glaucoma remains a contested issue. We aimed to determine whether neuroinflammation and immune infiltration were common, pervasive features of glaucoma by utilizing a unique resource of enucleated eyes from the archives of the St. Erik Ophthalmic Pathology Laboratory. The Ophthalmic Pathology archive at St. Erik Eye Hospital in Stockholm, Sweden includes ~ 3000 enucleated eyes preserved as paraffin wax tissue blocks with clinical records available for all patient eyes enucleated from 1960. Eye enucleations are performed due to ocular tumors, trauma, infection, and painful unresponsive glaucoma. Eyes are immediately put into 4% formalin following enucleation and wax embedded within 24 to 48 h. With national access to Sweden’s patient records, this represents a unique resource for glaucoma pathophysiology discovery.

## Materials and methods

### Ethics

Access to histopathology archive samples was fully covered through biobank #366 (St. Erik Eye Hospital). The study adhered to the tenets of the Declaration of Helsinki and the ethics protocols were approved by the Swedish Ethical Review Authority (2020-01525 and 2021-01036). This research followed the tenets of the Declaration of Helsinki.

### Sample selection

Enucleation reports from the histopathology archive were screened to identify suitable eyes. Inclusion criteria included eyes where the majority of retina and entire optic nerve head was preserved without severe atrophy. Exclusion criteria included ongoing infection at time of enucleation, a mass (i.e. tumor, hemorrhage) covering central retina, and in vivo detached retina (i.e. as opposed to a processing artefact). Specific additional inclusion criteria for glaucoma eyes included eyes previously diagnosed with glaucoma or ocular hypertensive eyes or showing clinical signs of glaucoma at time of enucleation. Exclusion criteria were descriptions of complete retinal nerve fiber layer (RNFL) or inner retinal atrophy. Existing archived sections of selected eyes were evaluated by light-microscopy for complete optic disk excavation, complete degeneration of the ganglion cell layer (GCL) and/or RNFL, or complete optic nerve atrophy. Since healthy eyes are not enucleated, eyes with uveal melanoma where the tumor did not infringe on the retina or optic nerve were used as controls (*e.g.* tumor confined to the iris and/or ciliary body and/or anterior choroid). Controls were selected by age and year of enucleation matching. Patients details are summarized in Table 1.

**Table 1:** Clinical characteristics of glaucoma and control donors

Sample ID	Eye	Condition	Sex	Age at enucleation (years)	Year of enucleation	Visual acuity (BCVA)	IOP (mmHg)	Years with glaucoma diagnosis
E0152-06	R	Glaucoma (unspecified)	F	87	2006	Amaurosis	57	23
E0554-05	R	Glaucoma (PEX)	F	82	2005	Amaurosis	50	13
E0048-04	R	Glaucoma (PEX)	F	86	2004	Amaurosis	48	~ 30
E0568-04	L	Glaucoma (neovascular)	F	77	2004	Amaurosis	50	Unknown
E0472-03	L	Glaucoma (unspecified)	F	73	2003	Amaurosis	25	Unknown
E0281-04	L	Glaucoma (POAG)	F	89	2004	Amaurosis	Last IOP not recorded	Unknown
E0469-04	R	Glaucoma (unspecified)	F	56	2004	Amaurosis	Last IOP not recorded	Unknown
E0599-02	R	Glaucoma (unknown)	M	34	2003	Amaurosis	Last IOP not recorded	Unknown
E0245-07	R	Control	M	86	2007	0.2	13	NA
E0029-06	L	Control	M	71	2006	NA	12	NA
E0300-06	R	Control	F	71	2006	0.1	14	NA
E0258-05	L	Control	F	67	2005	1	14	NA
E0330-05	R	Control	M	85	2005	0.2	12	NA
E0024-04	R	Control	M	61	2004	0.3	10	NA
E0271-02	R	Control	F	69	2002	1	17	NA
E0169-01	L	Control	M	72	2001	0.2	11	NA
E0214-01	R	Control	F	77	2001	HR	23	NA
E0588-01	R	Control	F	66	2001	FC 3 m	16	NA
E0001-00	R	Control	F	87	2000	Not recorded	Not recorded	NA
E0471-00	Unknown	Control	M	61	2000	R: 0,8 L: 1,0	Right: 16 Left: 17	NA

Sample ID	Medications	Other diseases	Optic Nerve Status at Enucleation	Chamber angle/Iris Status at Enucleation
E0152-06	Cyanocobalamin (Vitamin B). Acetylsalicylic acid (Trombocyte aggregation inhibitor). Furosemid (Loop-diuretic). Risperidone (Neurolepticum). Alprazolam (Benzodiazepine). Zopiclone (pyrazolopyrimidine). Propiomazin. Citalopram (SSRI). Mirtazapine (anti-depressive). Donepezil (Cholinesterase inhibitor). Lactulos (Laxitive). Hydroxizin. Paraffin, vaselin (*Oculentum simplex*)	Hypertension. Depression	Excavation and atrophy	Atrophy. Neovascularization. Occluded chamber angle
E0554-05	Pravastatin (Statin). Sotalol (Beta receptor blocker). Klopidogrel (Trombocyte aggregation inhibitor). Digoxin. Furosemid (Loop diuretic). Nitroglycerin. Indometacin (NSAID). Lansoprazole (Proton pump inhibitor). Lactitol (laxative)	Previous gall bladder operation. Hysterectomy and removal of ovaries. Heart infarction 1995. Angina. Heart failure. Cataract	Excavation and atrophy	Neovasculation. Mostly occluded chamber angle
E0048-04	Diabetes medication. Irbesartan (Angiotensin II antagonist). Furosemid (Loop diuretic). Verapamil (Calcium antagonist). Digoxin. Allopurinol (xanthine oxidase inhibitor). Anastrozole (Nonsteroidal aromatase inhibitor). Diclofenac (NSAID). Propiomazine (phenothiazines)	Diabetes Type II. Hypertension. Gout. Breat cancer operstion in 2003	Excavation and atrophy	Atrophy. Synechiae and neovascularization of chamber angle
E0568-04	Glibenclamide (Sulfonylurea). Candesartan (angiotensin receptor antaonist). Paracetamol (Acetaminophen). C-vitamin. Estradiol (Estrogen). Insulin (*Actrapid, Mixtard, and Insulatard)*	Neovascular glaucoma. Previous cholecystectomy. Hypertension. Diabetes. Mild dementia. Incontinence	Excavation and atrophy	Anterior synechiae. Neovascularization of chamber angle
E0472-03	Latanoprost (Prostaglandin analog). Timolol (beta blocker). Estradiol, Norethisterone (Estrogen, Progestin)	Glaucoma and cataract left eye for several years. Otherwise healthy	Excavation and atrophy	Chamber anlge partially occluded with anterior synechiae and neovascularization. Some iris neovascularization
E0281-04	Unknown	No other diseases recorded	Significant papillary excavation. Neovascularization and atrophy of opticus	Atrophy with neovascularization of iris and neovascularization of chamber angle
E0469-04	Unknown	Macular degeneration. Cataract	Excavation and atrophy	Some atrophy. Open chamber angle. No neovascularization
E0599-02	Unknown	Chronic uveitis	Excavation and atrophy	Atrophy. Occluded chamber anlge with anterior synechiae and neovascularization
E0245-07	Bendroflumethiazide, Potassium chloride (Diuretic, electrolyte). Enalapril (ACE inhibitor). Atorvastatin (statin). Acetylsalicylic acid (Trombocyte aggregation inhibitor). Nitroglycerin	Operated prostate in 1983. Hypertension. Heart infarction in 2004. Angina	No atrophy	Normal for age
E0029-06	Enalapril (ACE inhibitor). Felodipine (Calcium antagonist). Bendroflumethiazide, Potassium chloride (Diuretic, electrolyte). Acetylsalicylic acid (Trombocyte aggregation inhibitor)	Operated prostate in 1997. Hip surgery for left hip × 2 with most recent surgery in 2004. Knee surgery for right knee in 2003. Stroke in 2003, since then hemiparesis with partiall recovery	No atrophy	Tumor invasion. Chamber angle partially occluded by tumor
E0300-06	Levothyroxine (thyroid hormone). Losartan (Angiotensin II receptor antagonist).Furosemid (Loop diuretic). Cyanocobalamin (Vitamin B). Estradiol (Estragen). Amlodipine (Calcium antagonist)	No other diseases recorded	No atrophy	Tumor invasion
E0258-05	Amlodipine (Calcium antagonist). Ranitidine (Anti-histamine)	Hypertension	No atrophy	Normal for age
E0330-05	Diclofenac (NSAID)	Previously operated × 3 for kidney stones. Gout	No atrophy	Normal for age
E0024-04	No medications	Appendectomized	No atrophy	Tumor invasion of the iris
E0271-02	No medications	Healthy. Was admitted to a hospital many years prior due to a benign gynecological event	No atrophy	Normal for age
E0169-01	No medications	Previously operated for mitralis insufficiency in April 2000 with plastic ring. Pacemaker since fall 2000	No atrophy	Normal for age
E0214-01	No medications	Healthy. No previous operations	No atrophy	NA
E0588-01	Amitriptylin (tricyclic antidepressant). Atenolol (Beta receptor blocker). Estradiol (Estrogen)	Recurring depression	No atrophy	Tumor invasion
E0001-00	Unknown	No other diseases recorded	No atrophy	Normal for age
E0471-00	No medications	Previously healthy	No atrophy	Normal for age

### Immunohistochemistry

New ONH sections were cut from selected samples using a wax microtome (Thermo Scientific Microm HM355S). Tissue was sectioned at a thickness of 3 μm, placed on glass slides, and baked at 60 °C for 1 h. Chromogenic immunohistochemistry was performed using a Bond-III fully automated IHC and in situ hybridization machine (Leica Biosystems Division of Leica Microsystems Inc., Buffalo Grove, IL). Sections were deparaffinization in Bond DeWax solution followed by rehydration through an ethanol gradient. Antigen retrieval was performed by immersing in 1 mM EDTA buffer (pH 8.9–9.1) for 30 min at 100 °C. The sections were then washed and incubated in primary antibody for 15 min (Table 2). Slides were washed again and incubated in a polymer conjugated secondary antibody (BOND Polymer Refine Red Detection or BOND Polymer Refine Detection (DAB chromogen)) for 50 min at room temperature followed by color development for 15 min. Slides were counterstained with hematoxylin. Sections were dehydrated through an ethanol gradient, cleared in xylene, mounted in PERTEX, and covered with a coverslip.

**Table 2:** Antibody details

Antibody	Supplier	Ref. NR	Host	Dilution	Chromogen
RBPMS	Novus Biologicals	NBP2-2011	Rabbit	1:400	BOND Polymer Refine Red
NF-M	Novus Biologicals	NB300-222	Chick	1:2000	BOND Polymer Refine Detection (DAB)
CD45	DAKO	M0701	Mouse	1:100	BOND Polymer Refine Red
CD68	DAKO	M 0876	Mouse	1:100	BOND Polymer Refine Red
CD163	Abcam	ab182422	Rabbit	1:200	BOND Polymer Refine Red
IBA1	Abcam	ab178846	Rabbit	1:500	BOND Polymer Refine Red
GFAP	Abcam	ab4648	Mouse	1:1000	BOND Polymer Refine Red
Vimentin	Dako	M0725	Mouse	1:1000	BOND Polymer Refine Red

### Image acquisition and analysis

Tiled, color images of the whole retina and optic nerve were acquired at 40X magnification using a ZEISS Axio Scan.Z1 Digital Slide Scanner (Carl Zeiss). Regions of retina were cropped for analysis at 2 mm and 6 mm either side of the ONH center using Zen lite Software (version 3.3; Carl Zeiss). Crops of 1 mm were exported as Tiff Format (64bit, Big Tiff) images for analysis in FIJI. The ONH was exported as crops of 500 µm thickness starting from the optic disk surface and proceeding distally up to 2 mm. RBPMS + cells within the GCL were counted in crops (at 2 mm and 6 mm) in FIJI [31] and expressed as mean ± SEM. For immune cell counts, counts were performed manually along the entire retina within Zen lite. Cells were counted within the following retinal layers: ganglion cell complex (GCC, comprising the retinal nerve fiber layer, ganglion cell layer, and inner plexiform layer), inner nuclear layer (INL), outer plexiform layer (OPL), and outer nuclear layer (ONL). Cell counts were normalized to 100 µm of retina by measuring the retinal length along the ONL outer border in FIJI. For semi-quantitative analysis of signal intensity, crops of retina or ONH were color deconvoluted using a vector designed for DAB, FastBlue, and FastRed in FIJI. The channel corresponding to FastRed or DAB (depending on which stain was used) was retained (Mixed Red Refine, as used here, is the same chromogen as FastRed). This separated the signal of the chromogen targeted to the antibody from hematoxylin. The mean grey value (mean pixel intensity), and layer thickness were measured. Mean pixel intensity was inverted (value subtracted from 255) so that higher values corresponded to higher signal intensity. To determine the area of retina or ONH occupied by labelling, cropped images were binarized and particle analysis was performed in FIJI (Size: 0-Infinity; Circularity: 0–1) to give % area covered. Retinal values at 2 mm and 6 mm were averaged between either side of the ONH in glaucoma cases.

### Statistical analysis

Statistical analyses were performed in R. *Shapiro–Wilk* tests confirmed that data were not normally distributed and so comparisons were made using non-parametric tests. For comparison within individual retina (i.e. paired test as for comparison of retina adjacent to the tumor and opposite the tumor in controls) a *Wilcoxon signed-rank* test was used. For comparison of control and glaucoma samples a *Mann–Whitney U* test was used. A statistically significant difference was defined as *P* < 0.05 and categorized for graphics as * = P < 0.05, ** = *P* < 0.01, ***P < 0.001, NS = non-significant (*P* > 0.05). For box plots, the center hinge represents the median with upper and lower hinges representing the first and third quartiles; whiskers represent 1.5 times the interquartile range.

## Results

### Significant retinal ganglion cell loss in late-stage glaucoma

**Fig. 1:**
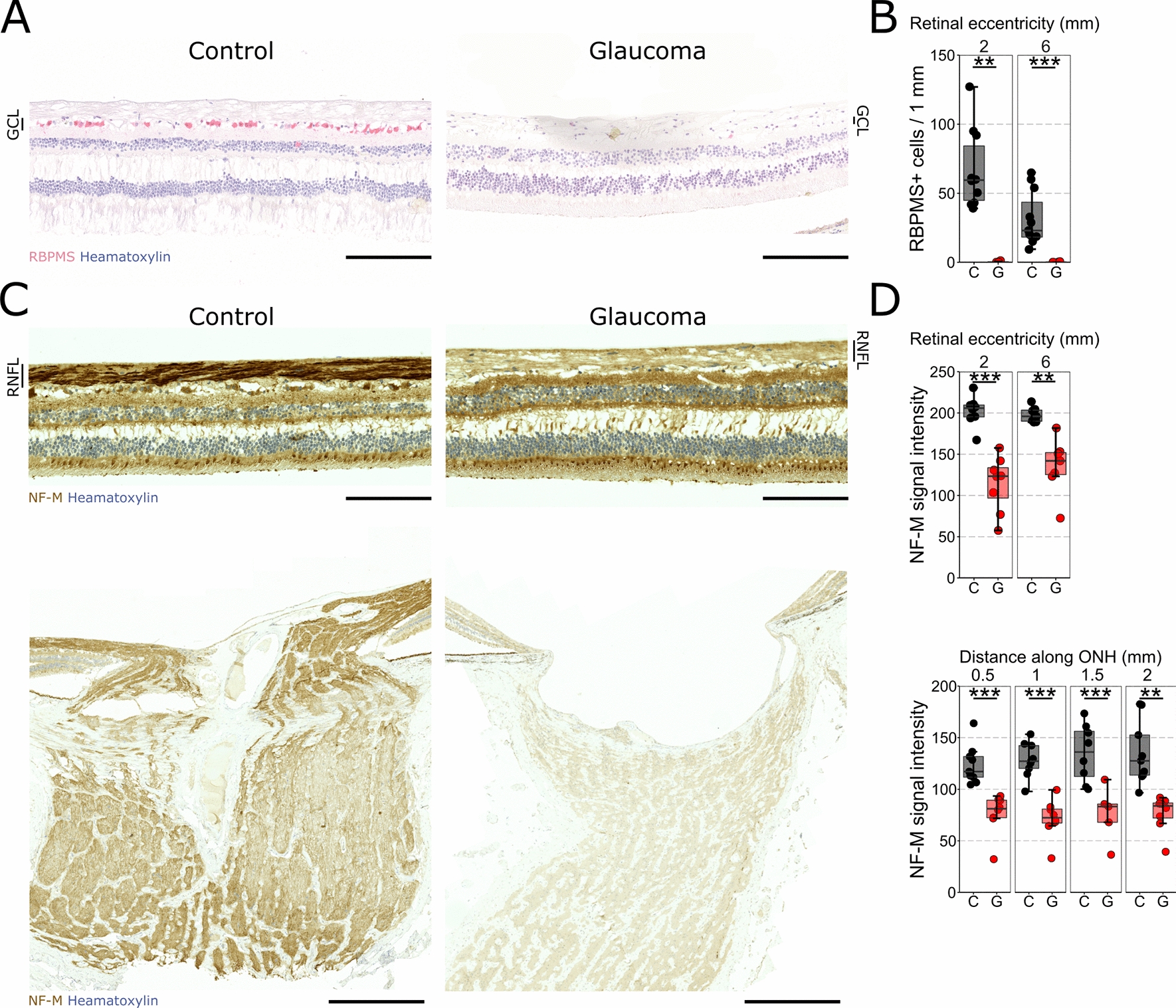
Significant retinal ganglion cell loss has occurred in late-stage glaucoma. **A** Control and glaucomatous retinas were labelled for RBPMS (red) to determine RGC density. 40X tiled images were cropped to 1000 µm of retina at 2 mm (central) and 6 mm (peripheral). **B** RGC density was significantly reduced in both central and peripheral retina with an almost complete absence of RGCs in the glaucoma samples. **C** Axon density was estimated by NF-M staining in the RNFL in retina and in the optic nerve head (brown). **D** Signal intensity was significantly reduced in the RNFL in both central and peripheral retina and along the whole ONH suggesting a significant loss of axon density. These suggest that all glaucoma samples are at a late-disease stage with significant retinal ganglion cell loss. Scale bar = 200 µm in A and C (retina), 500 µm in C (ONH). ** = *P* < 0.01, *** = *P* < 0.001. C = Control, G = Glaucoma. RBPMS = RNA-binding protein with multiple splicing, RGC = retinal ganglion cell, NF-M = Neurofilament medium, RNFL = retinal nerve fiber layer, ONH = optic nerve head

We determined the extent of RGC loss in selected donor eyes with advanced vision loss and/or amaurosis. In controls we identified 67 (± 9) and 31 (± 6) RGCs/mm of retina at 2 and 6 mm eccentricity from the ONH respectively, but < 1 RGC/mm in glaucomatous retina (0.25 ± 0.2 and 0.08 ± 0.08 at 2 and 6 mm) (Fig. 1A, B). Since single sections through the ONH may overestimate RGC loss due to a limited area of sampling we also stained NF-M in the RNFL. NF-M signal intensity was significantly reduced in the RNFL at 2 mm (44% relative to control) and 6 mm (32% relative to control) eccentricity and along the whole distance of the ONH (~ 40–45% relative to control) (Fig. 1C, D) demonstrating significant loss of RGC axons but with more remaining neural content than RBPMS counts alone would suggest.

### No detectable active monocyte infiltration in late-stage glaucoma

**Fig. 2:**
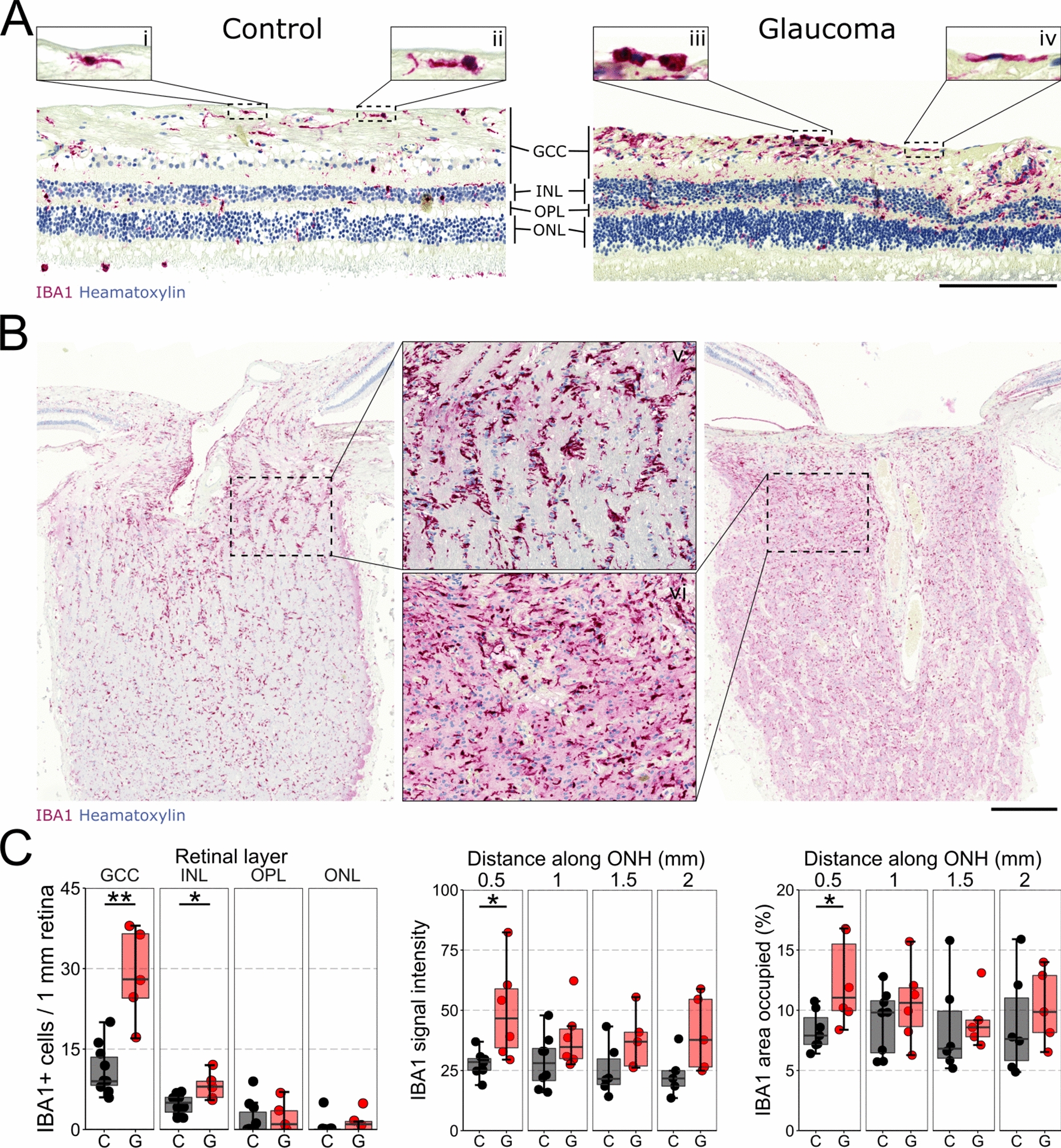
Microglial and monocyte morphology and density are increased in glaucoma, consistent with increased neuroinflammatory activation. Control and glaucomatous retinas were labelled for IBA1 (red) to visualize microglia/macrophages. **A** Whole retina (tiled 40X images) and **B** ONH were imaged and analyzed. (**C**) IBA1+ cells were counted by retinal layer across the whole retinal length, demonstrating a significant increase in cell density in the GCC and INL, but not in the OPL or ONL, consistent with inner retinal inflammation. The difference in IBA1+ cell morphology between control and glaucoma samples is highlighted in inset i-iv in **A**. IBA1+ cells in control retina demonstrate ramified processes (i, ii) consistent with homeostatic resting microglia. In comparison, in glaucoma IBA1+ cells have an amoeboid appearance (iii) consistent with pro-inflammatory phenotypes, or spindle/bipolar phenotypes (iv) at the RNFL/ILM border as are typical in animal models of glaucoma. Microglia/macrophages in control ONH (v) were restricted to areas between axon bundles but this spatial distribution was lost in glaucoma with microglia spread across the whole ONH suggesting proliferation or migration to areas of inflammation. This could also reflect the aftereffects of monocyte infiltration. In the ONH, microglial density and signal intensity was significantly increased within the first 500 µm, suggesting the presence of pro-inflammatory response in the laminar region. Scale bar = 200 µm in **A**, 500 µm in **B**. * = *P* < 0.05, ** = *P* < 0.01. C = Control, G = Glaucoma. IBA1 = ionized calcium-binding adapter molecule 1, ONH = optic nerve head, GCL = ganglion cell layer, INL = inner nuclear layer, OPL = outer plexiform layer, ONL = outer nuclear layer, RNFL = retinal nerve fiber layer, ILM = inner limiting membrane

Data from animal models suggests significant monocyte infiltration during early and late ocular hypertensive events and/or proliferation of microglia within inflamed tissue [8, 9]. However, it is currently unknown as to whether this occurs in human glaucoma. To assess the presence and degree of circulating monocyte entry into the retina and optic nerve in glaucoma we compared labelling of microglial, monocyte, and pan-leukocyte markers. Since control tissue were cases of ocular tumors, we first established the impact of a retinal tumor on inflammation and immune infiltration. As expected, tumors were immune cell rich and the retina on the side of the tumor had significantly higher numbers of immune cells (4.3 fold greater number of CD45+, 2.4 fold greater number of CD163+, 1.9 fold greater number of IBA1+) in comparison to the opposite side of the retina (Additional file 1: Fig. S1). We subsequently used only the retina opposite the tumor for comparisons of glaucoma to controls. In control and glaucoma retina, there were few cells positive for the pan-leukocyte marker CD45, and this was not significantly different between groups (Additional file 2: Fig. S2A–C). This was similar in the ONH, where signal intensity and the percentage of the area of the ONH occupied by staining was low (< 1% area) (Additional file 2: Fig. S2B–C). This trend was similar for the monocyte marker CD163. Few CD163 + cells per mm of retina were detected and signal intensity and area occupied were low in the ONH (< 1% area). There was no significant difference between control and glaucoma (Additional file 2: Fig. S2D–F). Labelling with the microglia/macrophage-specific marker IBA1 identified a greater number of cells in the retina and ONH (Fig. 2). IBA1+ cells in controls displayed ramified morphology typical of normal surveilling microglia. In glaucomatous retina, IBA1+ cells were more condensed around the nucleus as is typical of microglia which have shifted to a pro-inflammatory phenotype. There were numerous amoeboid IBA1+ cells typical of pro-inflammatory, pro-phagocytic microglia, or infiltrating monocytes. These cells were concentrated towards the inner border of the ILM. In addition, rod-like/bipolar-like IBA1+ cells were also concentrated at the inner border of the ILM. These are reminiscent of microglia observed in the NFL of animal models of glaucoma which align towards the ONH. IBA1+ cell density increased in the GC complex (260% of control) and INL (170% of control) but not in the ONL and OPL in glaucoma, consistent with neuroinflammation of only the inner retina mirroring neurodegeneration of only the inner retina as is observed in glaucoma (Fig. 2A–C). Both signal intensity (> 25 AU) and area occupied (~ 10%) in the ONH were higher for IBA1 staining than CD45 and CD163. There was a significant increase in both signal intensity (178% of control) and area occupied (148% of control) within the first 500 µm of the ONH in glaucoma (Fig. 2B, C), suggesting an increase in inflammation in the pre-myelinated optic nerve.

### Retina and optic nerve are extensively gliotic in late-stage glaucoma

**Fig. 3:**
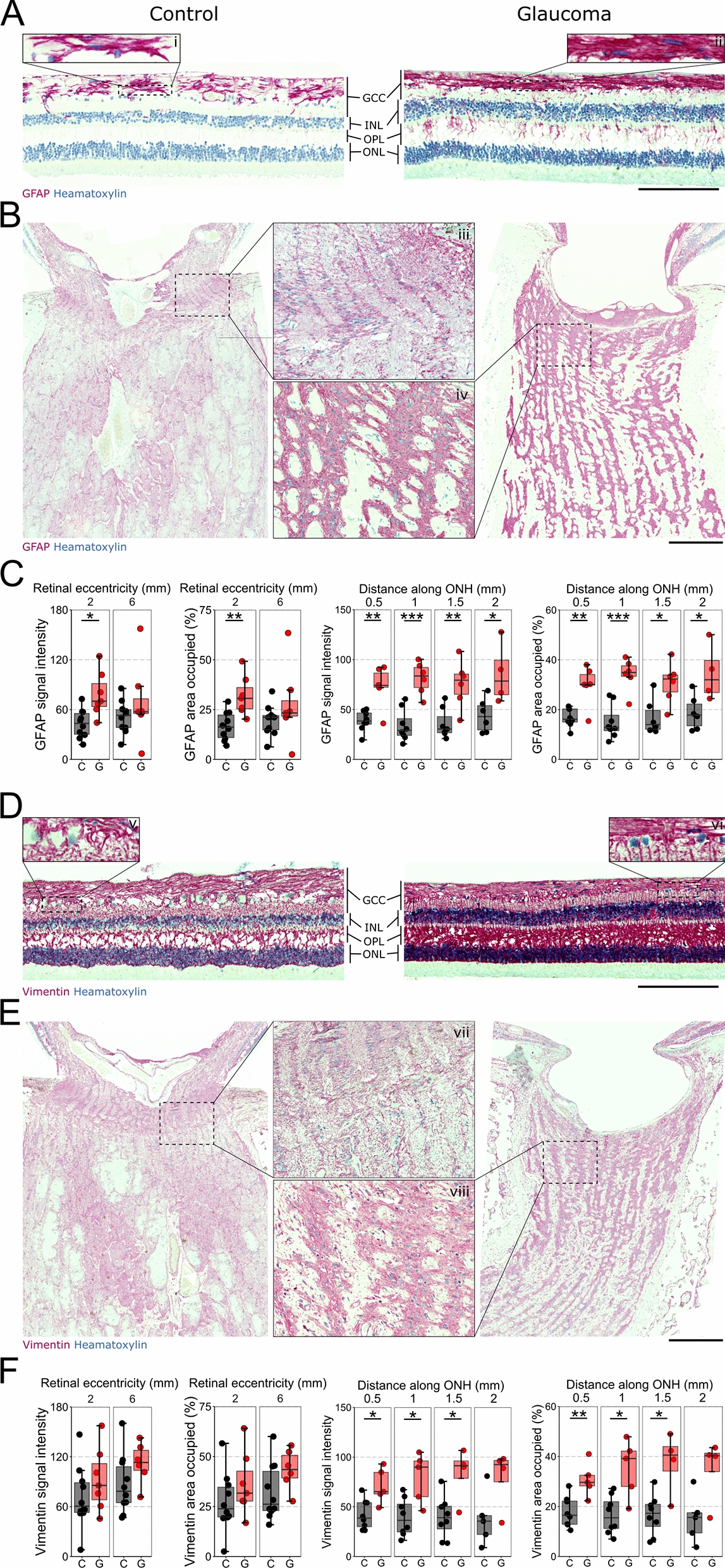
Significant gliosis has occurred in late-stage glaucoma. Control and glaucomatous retinas were labelled for GFAP (red) to visualize astrocytes in **A** the retina and **B** the ONH. Astrocytes in control retinas (i) had clearly defined processes (typical of stellate morphology) whereas in glaucoma samples (ii) there was clear expansion of GFAP content such that individual cell morphology was obscured. GFAP staining was evident in the OPL and IPL, consistent with Müller glia upregulation of GFAP. Astrocytes in control ONH (iii) were well organized such that they clearly delineate the areas between axon bundles whereas in glaucoma (iii) there was a hypertrophy of astrocytes typical of gliosis and neural loss. **C** GFAP signal intensity was significantly greater in the RNFL in glaucoma in central but not peripheral retina, as was the area of RNFL occupied by GFAP labelling. **D** retina and **E** ONH were also labelled with vimentin (red) to identify astrocytes and Müller glia. Strong labelling for vimentin was achieved in both control and glaucoma retina across all layers highlighting Müller glia processes. In the IPL of control retina (v) these processes are less ordered, reflecting their association with synapses, whereas in glaucoma (vi) the IPL demonstrates many longitudinal fibers running through the IPL as is common in pro-inflammatory, reactive Müller glia. In the ONH vimentin labelling of astrocytes in control (vii) and glaucoma (viii) demonstrated the same trend as with GFAP. **F** Vimentin labelling in the retina was variable and there was no significant difference in signal intensity or area occupied between control and glaucoma. In the ONH, vimentin signal intensity and area occupied were significantly increased in glaucoma relative to control across the first 1.5 mm of ONH, further supporting astrocytic gliosis in the ONH. Scale bar = 200 µm in **A**, **D**, 500 µm in **B**, **E**. * = *P* < 0.05, ** = *P* < 0.01, *** = *P* < 0.001. C = Control, G = Glaucoma. GFAP = Glial fibrillary acidic protein, ONH = optic nerve head, OPL = outer plexiform layer, RNFL = retinal nerve fiber layer, IPL = inner plexiform layer

GFAP and vimentin are type III intermediate filament proteins that are upregulated in astrocytes and Müller glia in response to inflammatory activation [32]. Intense GFAP and vimentin staining was observed across the retina and ONH in both controls and glaucoma (Fig. 3A–F). GFAP signal intensity (183% of control) and area occupied (190% of control) was significantly higher in glaucoma at 2 mm eccentricity but not at 6 mm (Fig. 3A–C). Stellate astrocyte morphology was evident in the NFL in control but in glaucoma retinas there were no distinguishable individual cells, rather the network had become interwoven. GFAP expression was also evident within Müller processes (transverse along retinal layers). These are clear indications of significant gliosis. In the ONH, GFAP signal intensity (~ 200% of control on average) and area occupied (~ 190% of control on average) was significantly higher in glaucoma along the whole distance of the ONH (Fig. 3B, C). Vimentin was variable in both conditions in the retina and was not significantly different (Fig. 3D–F) but transverse Müller processes were again more evident. Vimentin was significantly greater in the ONH in glaucoma, with higher signal intensity (~ 195% of control on average) and area occupied (~ 200% of control on average) up to 1.5 mm along the ONH (Fig. 3E, F). These demonstrate significant gliosis of the retina and ONH in glaucoma, consistent with previous inflammatory insult.

## Discussion

We identified significant RGC loss in glaucomatous samples consistent with late-stage disease. We identified a significant increase in density and volume of microglia/macrophages and gliosis in the retina and ONH in these glaucomatous eyes but with no evidence of actively infiltrating monocytes (findings summarized in Table 3).

**Table 3:** Qualitative summary of staining

Marker	Condition	CD45	CD168	IBA1	GFAP	Vimentin
Retina	Control	Low	Low	+	+	++
	Glaucoma	Low	Low	+++	++	++
ONH	Control	Low	Low	++	+	+
	Glaucoma	Low	Low	+++	++	++

Across multiple species and models of glaucoma, inflammation has been identified as a common feature of the disease. Importantly, inflammation in this context is not a bystander event, but is an active exacerbator of RGC neurodegeneration. Supporting this, strategies to prevent or limit inflammation have been demonstrated to provide robust protection of RGCs, extending the period of their viability. These treatments do not address intrinsic susceptibilities or dysfunctions of RGCs and so will not completely arrest neurodegeneration, but they could nevertheless prove to be ideal adjuvant treatments alongside existing IOP lowering therapies and new emerging strategies. Glaucoma shares many common features with other neurodegenerative diseases including Alzheimer’s disease and multiple sclerosis, where neuroinflammation is the prime therapeutic target [33]. Repurposing existing drugs or those which have passed safety trials but failed to show disease specific benefits, may represent a valuable strategy to deliver additional therapeutic benefits to glaucoma patients beyond conventional IOP lowering treatment. The major barrier to this is a lack of translational data supporting the occurrence of inflammatory events and mechanisms in human glaucoma as described in animal models. This is the result of a paucity of available and suitable human tissue. Since patients do not die from glaucoma it is difficult to acquire tissue at early disease stages where the quality of tissue is not confounded by the cause of death, or where there is not significant time post-mortem prior to tissue collection. We utilized enucleated eyes to diminish these issues. Since this tissue is not acquired post-mortem and is fixed immediately after enucleation, it is free of neurodegenerative and neuroinflammatory confounders that can be induced in degrading retina. We previously used post-mortem tissue from glaucoma patients fixed within ~ 2 h of death and experienced significant neural changes at a gross- and ultra-structural level [30]. Although the enucleated eyes used here were late/end-stage disease, identification of ongoing or past inflammation can be reliably attributed to the disease process.

We identified a significant increase in density and volume of microglia/macrophages and gliosis in the retina and ONH. We did not detect any specific labeling of actively infiltrating monocytes, but we cannot rule out the presence of integrated monocytes that have become microglia-like monocyte-derived macrophages. These infiltrating cells adopt phenotypes of resident cells, in the central nervous system this includes expression and upregulation of genes previously considered specific markers of resident microglia (*e.g.* IBA1). This process has now been well characterized and has been demonstrated in animal models of Alzheimer’s disease and retinal photoreceptor degeneration [34–37]. In animal models of glaucoma, both active extravasation of monocytes and passive entry of monocytes (leakage) has been demonstrated [8, 38] as well as blood-retina-barrier disruption [7, 39]. It is yet to be determined whether such blood-retina-barrier breakdown exists in human glaucoma patients, and as such, we cannot distinguish whether the immune cells that have entered have done so via an active or passive mechanism. Margeta et al*.*, previously demonstrated significant monocyte infiltration to the ONH in glaucoma [16]. This may occur as an earlier disease feature, as is observed in animal models of glaucoma, and by later disease stages this process may have ceased. It is unclear whether the cells present in this study have entered through via an active or passive mechanism. Further studies could distinguish whether blood-retina-barrier integrity in post-mortem human samples is disrupted. Alternatively, the post-mortem time of  > 10 h in all samples used by Margeta et al*.* may have resulted in significant monocyte infiltration or leakage into tissue post-mortem, which was further enhanced in glaucomatous eyes. This would not have occurred in our enucleated samples. It is possible the high levels of gliosis which we detected may present a barrier to further immune cell infiltration at later disease time points. Much of the lost neural content in the glaucoma cases was replaced by astrocyte and Müller processes in the inner retina and ONH. This will also prove a significant barrier to RGC replacement or regenerative therapeutic approaches.

### Strengths and limitations

Labelling for CD45 and CD163 was weak. This likely reflects the difficulty staining tissue that has been preserved in paraffin wax for  > 10 years. While some antibodies worked well with intense staining following antigen retrieval, CD45 and CD163 were not as pervasive as expected which may have resulted in underreporting of their abundance. Using more concentrated antibody resulted only in non-specific labelling and a difficulty in resolving cells against background signal. This further emphasizes the difficulty in confirming animal model findings in glaucoma human tissue. A potential weakness of this study is that glaucoma cases include a number of differing types of glaucoma (POAG, PEX, and unspecified). These could differ in the degree of inflammation, particularly if related to the degree of neuronal decline, and subdividing types would provide a more comprehensive analysis of inflammation given a greater number of available samples. Well characterized, and well-preserved glaucoma samples from earlier in disease will be necessary to fully assess inflammation at earlier disease time-points (however, access to this tissue is likely to be a major barrier). It is essential to elucidate early markers and mechanisms of inflammation in human samples if immunomodulatory therapies are to be successfully translated from animal studies. Animal models have demonstrated that strategies targeting neuroinflammation are highly time and context dependent. Attempting to prevent inflammation can be profoundly neuroprotective, and so an understanding of how early inflammation occurs will be important in determining which of these strategies can be clinically employed. Although this study only identifies inflammation as a feature of late disease, this at least supports that inflammatory events have occurred and/or are ongoing. These data do not rule out inflammation occurring only as a tombstone of the disease. However, if inflammation is only a byproduct of significant RGC degeneration, then this does not preclude anti-inflammatory therapies as viable strategies to limit acceleration of RGC degeneration much in the same way that current IOP lowering therapies do not target a causative disease feature. The main strength of this study is that the inflammatory changes identified are free from confounders related to the degree of tissue integrity and preservation.

## Conclusions

We identify evidence of widespread inflammation in the absence of post-mortem confounders in glaucomatous retina and optic nerve heads which support findings in animal models. These samples represent late/advanced disease stages; further evidence of early inflammation is critical to advance understanding of a potential role of inflammation in human glaucoma pathophysiology.

## Supplementary Information

**Additional file 1: Fig. S1.** Retina opposite the tumor has significantly less immune cell labelling than retina adjacent to the tumor in uveal melanoma eyes. Since healthy eyes are not enucleated, uveal melanoma cases where the tumor did not infringe on the central retina or optic nerve were used as controls. To determine the influence of the tumor on immune cells in the retina, cell counts of CD45+, CD16+, and IBA1+ cells were performed on retina each side of the ONH. Results were compared from the retina adjacent to the tumor (tumor side, TS) or opposite the tumor (opposite side, OS). **A** An example control eye showing the overview of the whole section where the tumor is visible in the upper right of the vitreous chamber and example images of IBA1+ cells from the tumor side (right) and opposite side (left). **B** There was a significantly greater number of CD45+, CD163+, and IBA1+ cells in the GCC on the tumor side relative to the opposite side. There was also a significant increase in IBA1+ cells in the INL on the tumor side relative to the opposite side. These data indicate that the opposite side represents the best approximation of normal retina and as such only the opposite side to the tumor was analyzed in all control eyes when comparing to glaucoma eyes. Scale bar = 5 mm in **A** (upper) and 200 µm in **A** (lower). * = *P* < 0.05, ** = *P* < 0.01. C = Control, G = Glaucoma. GCC = ganglion cell complex, IBA1 = ionized calcium-binding adapter molecule 1, INL = inner nuclear layer, ONH = optic nerve head, OS = opposite side.

**Additional file 2: Fig. S2.** Minimal labelling of CD45+ and CD163+ cells in control and glaucoma retina. Control and glaucomatous retinas were labelled for CD45 (red) to visualize leukocytes in **A** the retina and **B** the ONH. Few CD45+ cells were observed in either condition and these were predominantly observed in the GCC in both control (i) and glaucoma (ii) retinas. There were more CD45+ cells in the ONH but there was not clear order to their distribution (iii, iv). **C** Cell counts of CD45+ cells identified no significant difference in cell density between control and glaucoma across all retinal layers. In the ONH there was no significant difference in signal intensity or area occupied by labelling. Control and glaucomatous retinas were labelled for CD163 (red) to visualize macrophages in **A** the retina and **B** the ONH. As with CD45, there were few CD163+ cells in the retina and these were predominantly in the GCC in control (v) and glaucoma (vi). In the ONH there were few CD163+ cells with no clear order to their distribution (vii, viii). **F** There was no significant difference between control and glaucoma retina in the density of CD163+ cells in any retinal layer, and no significant difference in signal intensity or area occupied in the ONH. Scale bar = 200 µm in **A**, **D**, 500 µm in **B**, **E**. * = *P* < 0.05, ** = *P* < 0.01. ONH = optic nerve head, GCC = ganglion cell complex

## Data Availability

The datasets used and/or analyzed during the current study are available from the corresponding author on reasonable request.

## Abbreviations

GCC: Ganglion cell complex
GCL: Ganglion cell layer
GFAP: Glial fibrillary acidic protein
IBA1: Ionized calcium-binding adapter molecule 1
INL: Inner nuclear layer
IOP: Intraocular pressure
IPL: Inner plexiform layer
NF-M: Neurofilament medium
ONH: Optic nerve head
ONL: Outer nuclear layer
OPL: Outer plexiform layer
RBPMS: RNA-binding protein with multiple splicing
RGC: Retinal ganglion cells
RNFL: Retinal nerve fiber layer
