# Supplementary figures and images for: Widespread retina and optic nerve neuroinflammation in enucleated eyes from glaucoma patients
Source: Acta Neuropathol Commun. 2022 Aug 19;10:118. doi: 10.1186/s40478-022-01427-3 (PMC9392254; doi:10.1186/s40478-022-01427-3)

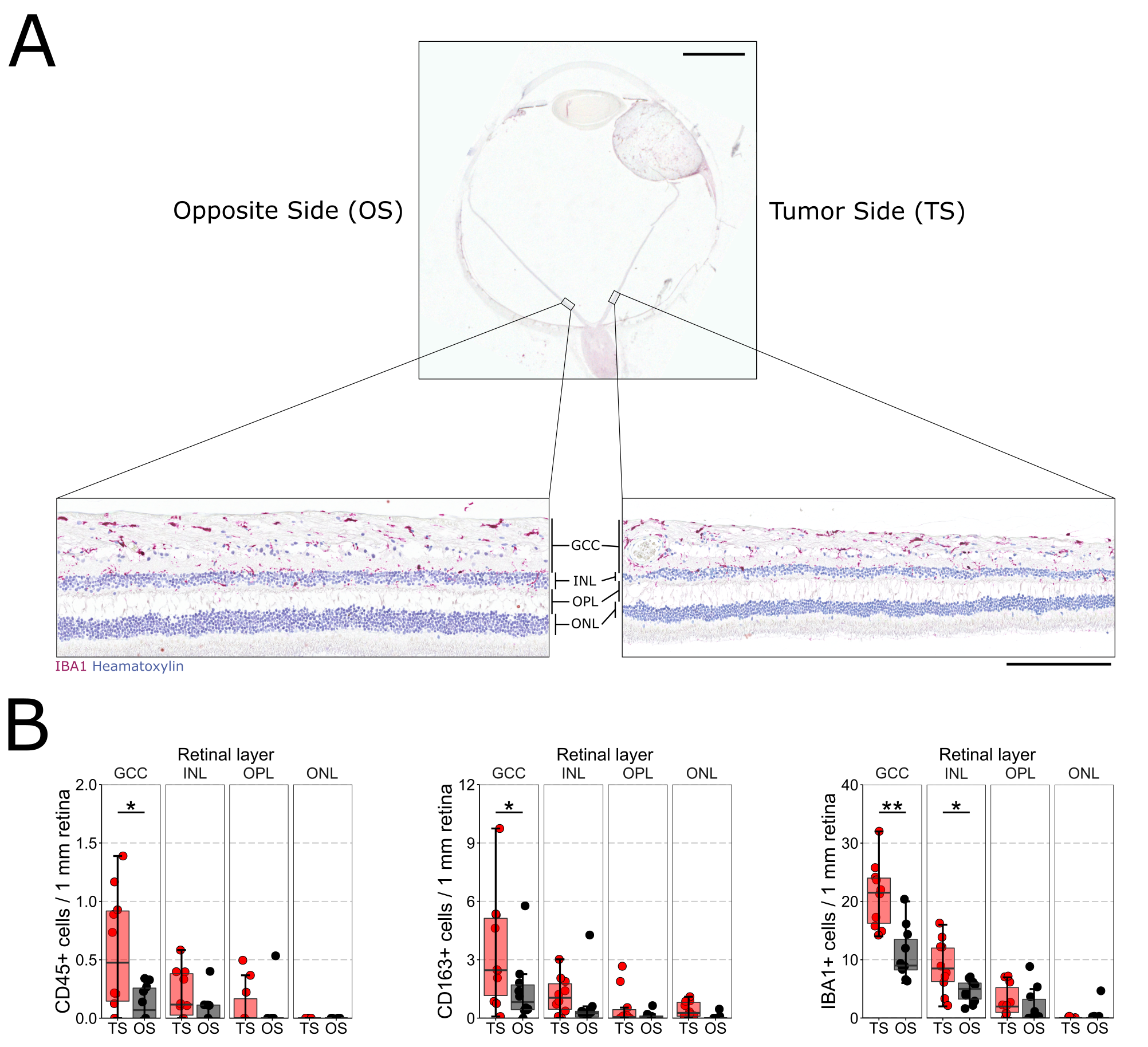

Supplement: Supplementary file 1 — Additional file 1: Fig. S1. Retina opposite the tumor has significantly less immune cell labelling than retina adjacent to the tumor in uveal melanoma eyes. Since healthy eyes are not enucleated, uveal melanoma cases where the tumor did not infringe on the central retina or optic nerve were used as controls. To determine the influence of the tumor on immune cells in the retina, cell counts of CD45+, CD16+, and IBA1+ cells were performed on retina each side of the ONH. Results were compared from the retina adjacent to the tumor (tumor side, TS) or opposite the tumor (opposite side, OS). A An example control eye showing the overview of the whole section where the tumor is visible in the upper right of the vitreous chamber and example images of IBA1+ cells from the tumor side (right) and opposite side (left). B There was a significantly greater number of CD45+, CD163+, and IBA1+ cells in the GCC on the tumor side relative to the opposite side. There was also a significant increase in IBA1+ cells in the INL on the tumor side relative to the opposite side. These data indicate that the opposite side represents the best approximation of normal retina and as such only the opposite side to the tumor was analyzed in all control eyes when comparing to glaucoma eyes. Scale bar = 5 mm in A (upper) and 200 µm in A (lower). * = P < 0.05, ** = P < 0.01. C = Control, G = Glaucoma. GCC = ganglion cell complex, IBA1 = ionized calcium-binding adapter molecule 1, INL = inner nuclear layer, ONH = optic nerve head, OS = opposite side. [file 40478_2022_1427_MOESM1_ESM.tif]

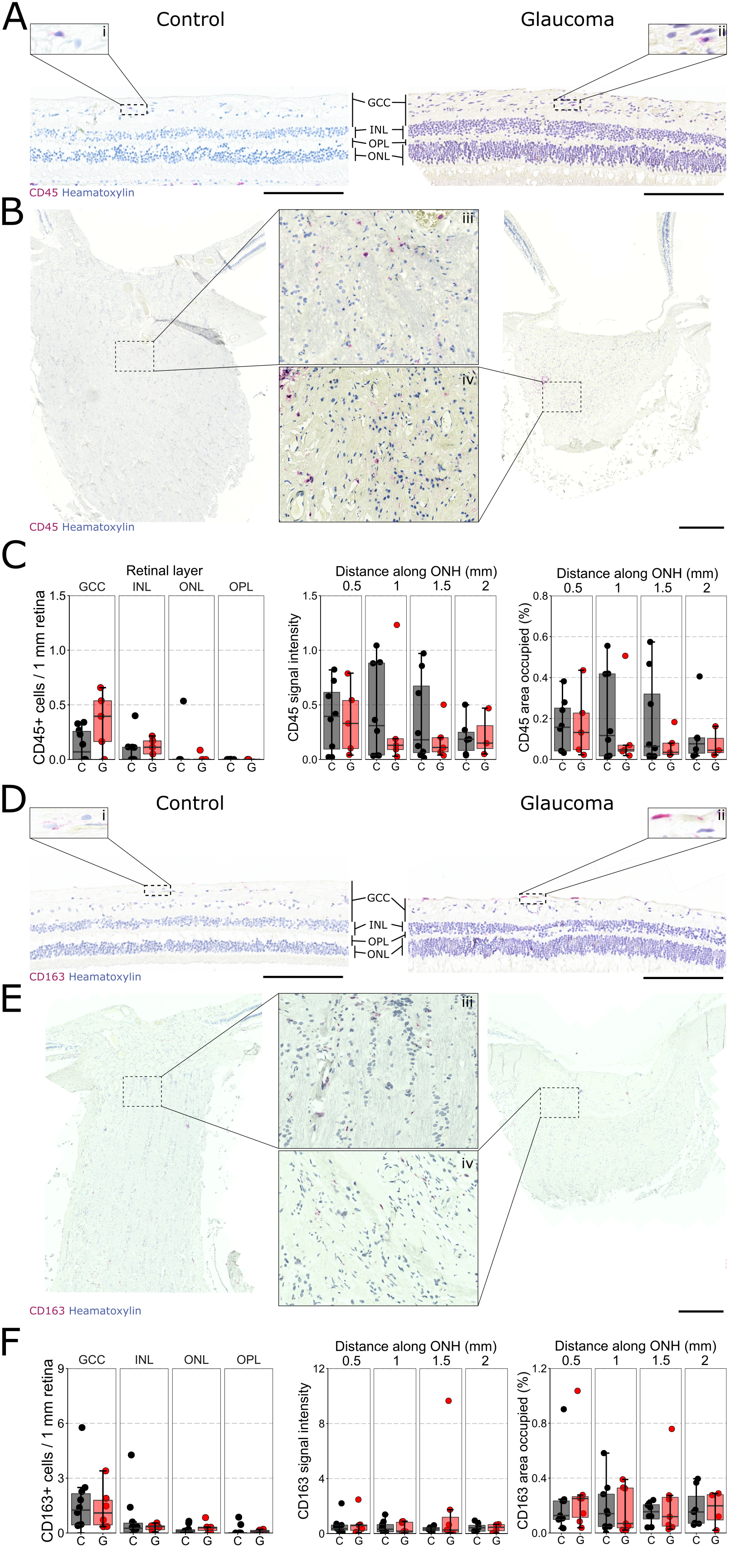

Supplement: Supplementary file 2 — Additional file 2: Fig. S2. Minimal labelling of CD45+ and CD163+ cells in control and glaucoma retina. Control and glaucomatous retinas were labelled for CD45 (red) to visualize leukocytes in A the retina and B the ONH. Few CD45+ cells were observed in either condition and these were predominantly observed in the GCC in both control (i) and glaucoma (ii) retinas. There were more CD45+ cells in the ONH but there was not clear order to their distribution (iii, iv). C Cell counts of CD45+ cells identified no significant difference in cell density between control and glaucoma across all retinal layers. In the ONH there was no significant difference in signal intensity or area occupied by labelling. Control and glaucomatous retinas were labelled for CD163 (red) to visualize macrophages in A the retina and B the ONH. As with CD45, there were few CD163+ cells in the retina and these were predominantly in the GCC in control (v) and glaucoma (vi). In the ONH there were few CD163+ cells with no clear order to their distribution (vii, viii). F There was no significant difference between control and glaucoma retina in the density of CD163+ cells in any retinal layer, and no significant difference in signal intensity or area occupied in the ONH. Scale bar = 200 µm in A, D, 500 µm in B, E. * = P < 0.05, ** = P < 0.01. ONH = optic nerve head, GCC = ganglion cell complex [file 40478_2022_1427_MOESM2_ESM.tif]
